# The effect of gantry spacing resolution on plan quality in a single modulated arc optimization

**DOI:** 10.1120/jacmp.v12i4.3603

**Published:** 2011-11-15

**Authors:** Ivaylo B. Mihaylov, Bruce Curran, Edward Sternick

**Affiliations:** ^1^ Department of Radiation Oncology Rhode Island Hospital/Brown Medical Center Providence RI 02903

**Keywords:** VMAT, arc, optimization, dose, IMRT, segments

## Abstract

Volumetric‐modulated arc technique (VMAT) is an efficient form of IMRT delivery. It is advantageous over conventional IMRT in terms of treatment delivery time. This study investigates the relation between the number of segments and plan quality in VMAT optimization for a single modulated arc. Five prostate, five lung, and five head‐and‐neck (HN) patient plans were studied retrospectively. For each case, four VMAT plans were generated. The plans differed only in the number of control points used in the optimization process. The control points were spaced 2°, 3°, 4°, and 6° apart, respectively. All of the optimization parameters were the same among the four schemes. The 2° spacing plan was used as a reference to which the other three plans were compared. The plan quality was assessed by comparison of dose indices (DIs) and generalized equivalent uniform doses (gEUDs) for targets and critical structures. All optimization schemes generated clinically acceptable plans. The differences between the majority of reference and compared DIs and gEUDs were within 3%. DIs and gEUDs which differed in excess of 3% corresponded to dose levels well below the organ tolerances. The DI and the gEUD differences increased with an increase in plan complexity from prostates to HNs. Optimization with gantry spacing resolution of 4° seems to be a very balanced alternative between plan quality and plan complexity.

PACS number: 87.55.de

## I. INTRODUCTION

Intensity‐modulated radiation therapy (IMRT) offers great flexibility in delivering highly‐conformal dose distributions to complex targets, while at the same time sparing the surrounding healthy tissue and anatomical structures. On a conventional linear accelerator, IMRT can be delivered either with a fixed gantry angle technique or with a volumetric‐modulated arc technique (VMAT). VMAT evolved from intensity‐modulated arc therapy.^(^ ^1^ ^)^ It allows simultaneous variation of dose rate, gantry speed, and multileaf collimator (MLC) segments.^(^ ^2^ ^)^ The technique promises some dosimetric benefits^(^ ^3^ ^)^ together with reduced treatment times^(^ ^1^ ^,^ ^2^ ^,^ ^4^ ^–^ ^6^ ^)^ which, in turn, may have significant radiobiological impact.^(^ ^7^ ^–^ ^9^ ^)^

IMRT plan optimization (forward or inverse) consists of determination of appropriate intensity maps which, when superimposed on each other, result in a dose distribution delivering therapeutic dose to tumor(s), at the same time sparing as much as possible the surrounding organs at risk (OARs). From this point of view, the difference between fixed gantry angle IMRT and VMAT^(^ ^10^ ^)^ is that, in the former, the intensity levels are clustered into several (usually 7 to 9) discrete directions (fixed gantry angles) while, in the later, the intensity levels are uniformly distributed along a full or a partial arc. In either case, the number of intensity levels controls the dose conformality and, therefore, the ability for dose painting and IMRT plan quality.

It has been shown^(^ ^11^ ^–^ ^13^ ^)^ that for fixed gantry angle IMRT, the increase of number of intensity levels beyond a critical value has very little effect on the plan quality and prolongs the treatment time. For VMAT however, the increase of available intensity levels,^(^ ^10^ ^,^ ^14^ ^)^ achieved by superposition of several modulated arcs, allowed better dose conformality than in a single modulated arc. One of the studies^(^ ^14^ ^)^ utilized two‐stage optimization process, where fluence maps optimization and their conversion into multileaf collimator (MLC) trajectories are separated. The other work utilized deliverable^(^ ^15^ ^–^ ^18^ ^)^ optimization, where the fluence‐map‐to MLC trajectories conversion is incorporated into the optimization process, thereby explicitly accounting for MLC leaf position weights.

The purpose of this study is to investigate the relation between the number of segments (gantry spacing resolution) and plan quality in inverse optimization for a single deliverable^(^ ^2^ ^)^ modulated arc.

## II. MATERIALS AND METHODS

### A. Patients

Fifteen patient plans were retrospectively studied. The dataset consisted of five prostate, five lung, and five head‐and‐neck (HN) cases. For each patient, planning target volume (PTV) was outlined by the attending physician. In addition, for the HN cases, intermediate risk lymph nodes were also outlined as targets. Rectum and bladder were outlined as OARs for the prostate cases. For the lung cases, the OARs were healthy lung, heart, and spinal cord. The HN OARs included brainstem, spinal cord, mandible, and both parotid glands (parotids). All of the abovementioned targets and OARs were used as dose‐escalation and dose‐limiting structures in the inverse VMAT optimization process.

### B. Treatment planning

The prescription for the prostate cases ranged form 76 Gy to 78 Gy to 95% of the PTV volume (dose index PTV $D_{95\%}$), subject to rectum and bladder doses of 75 Gy, 70 Gy, and 60 Gy to 15%, 25%, and 40% of the volumes, respectively. The prescriptions for the lung PTVs $D_{95\%}$ ranged from 64 Gy to 76 Gy, limited by spinal cord maximum dose of 50 Gy, doses of 30 Gy and 20 Gy to healthy lung dose indices (DIs) $D_{20\%}$ and $D_{30\%}$, respectively, as well as 45 Gy to heart DI $D_{33\%}$. The HN PTV $D_{95\%}$ ranged from 64 Gy to 76 Gy, limited by maximum cord and brainstem doses of 50 Gy, average doses to parotids of less than 24 Gy, as well as parotids DI $D_{50\%}$ of less than 30 Gy, and mandible DI $D_{10\%}$ of less than 60 Gy. In addition, each plan was deemed as clinically acceptable only if the standard deviation of the dose over the PTV was less than 3%,^(^ ^19^ ^)^ which in essence is dose uniformity requirement.

The treatment planning was performed with ${\text{Pinnacle}}^{3}$ (v. 9.100, Philips Radiation Oncology Systems, Fitchburg, WI) treatment planning system (TPS). The patient plans were optimized with Pinnacle SmartArc module^(^ ^2^ ^)^ for a Varian 21EX (Varian Medical Systems, Palo Alto, CA) linear accelerator, equipped with a 120 leaf Millennium MLC. Each plan utilized a single volumetric modulated arc with 6 MV photons. Four VMAT plans were generated for each patient case. The plans utilized the same optimization objectives, the same angular arc length, and the same dose grid with voxel size of $0.3\;\times \;0.3\;\times \;0.3\;{\text{cm}}^{3}$. The only difference among the four plans was in the gantry spacing resolution. Details on the arc parameters for the different plans are given in Table 1. The arc lengths for prostate and HN cases were set to be almost full arcs (358°), therefore utilizing the entire solution space for optimization. For the lung cases, 220° arcs were chosen such that a reasonable balance between arc length (in terms of adequate number of segments) and contralateral lung irradiation was achieved. The dose calculations were performed with ${\text{Pinnacle}}^{3}$ collapsed cone convolution superposition algorithm.^(^ ^20^ ^,^ ^21^ ^)^

**Table 1:** Arc optimization details. For each anatomical site, angular arc length together with the number of control points for four different gantry spacing resolutions, are summarized.

	*Prostate*	*Lung*	*HN*
Angular Arc Length	358°	220°	358°
# of control points [2°]	181	111	181
# of control points [3°]	121	75	121
# of control points [4°]	91	56	91
# of control points [6°]	61	38	61

The plans for each patient, optimized with the different angular gantry spacing resolution, were normalized such that 95% of the PTV received the same dose. In addition, standard deviation of the dose across the PTV of less than 3%^(^ ^19^ ^)^ was achieved. Thereby, target coverage was essentially the same for all four optimization schemes and will not be discussed further.

### C. Analysis

The dose distributions computed with the finest 2° gantry spacing resolution were used as a reference to which the dose distributions computed with the more coarse resolutions were compared. The metric used in the comparison was based on DIs.^(^ ^16^ ^)^ Briefly, DIs are points on the DVHs corresponding to certain fractional anatomical volume coverage (e.g., PTV ${\text{DI}}_{95\%}$ is the point on the PTV DVH corresponding the 95% of the PTV volume). In the prostate cases, the evaluated DIs were PTV $D_{95\%}$, rectum and bladder $D_{15\%}$, $D_{25\%}$, and $D_{40\%}$. For the lung cases, the evaluated DIs were PTV $D_{95\%}$, spinal cord $D_{1\%}$, heart $D_{33\%}$, and healthy lung $D_{20\%}$ and $D_{30\%}$. For the HN cases, the evaluated DIs comprised PTV $D_{95\%}$, cord and brainstem $D_{1\%}$, and left and right parotid $D_{50\%}$. All of these DIs were also utilized in the inverse optimization process. The DIs computed from the dose distributions achieved with 2° separation were used as a reference to which the DIs obtained from the more coarse gantry spacing resolutions were compared. The compared DIs were normalized to the reference DIs. This normalization eases the comparison, especially as in the studied case, where different patients have different prescription doses^(^ ^16^ ^)^ and different OAR DIs, respectively. As a result, if a normalized DI has a value of one, it means that the compared DIs are equal; where as, if the value of the index is greater than one, it means that the compared DI is greater than the reference DI and smaller otherwise.

In addition to the DIs, generalized equivalent uniform doses^(^ ^22^ ^,^ ^23^ ^)^ (gEUDs) were also used in plan quality assessment. The “α‐values” for targets, rectum/bladder, lung, spinal cord, heart, brainstem, and parotids were set to −10, 6, 1.2, 7.4, 3.1, 4.6, and 5.0, respectively.^(^ ^24^ ^,^ ^25^ ^)^

## III. RESULTS

The results for the OARs from prostate, lung, and HN VMAT optimizations are presented sequentially below. Patients one to five are prostate cases, patients six to 10 correspond to the lung cases, and patients 11 to 15 are the HN cases.

### A. Prostate plans

**Figure 1:**
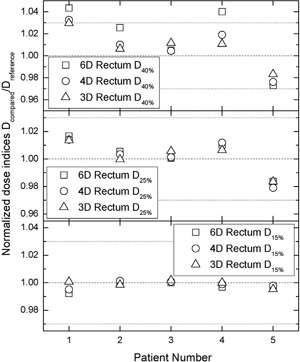
Comparison of normalized DIs for rectum. The normalization was performed with respect to the DIs derived from optimization scheme with 2° angular spacing between adjacent control points. The open squares, circles, and triangles represent the normalized DIs for angular spacing of 6°, 4°, and 3°, respectively (denoted as 6D, 4D, and 3D in the legend). The top, middle, and bottom panels outline the comparisons for rectum $D_{40\%}$, $D_{25\%}$, and $D_{15\%}$, respectively. The “one” level together with ±3% levels are denoted in each panel by a dashed or dotted lines, respectively.

The results for the rectum DIs, $D_{15\%}$, $D_{25\%}$, $D_{40\%}$ are presented in the bottom, middle, and top panels of Fig. 1, respectively. With very few exceptions, the difference among the reference and the compared DIs is within ±3%, which has been used as a surrogate for clinical significance.^(^ ^26^ ^–^ ^28^ ^)^ The results for the bladder are quite similar to the rectum results and are not presented in a graphical format.

**Figure 2:**
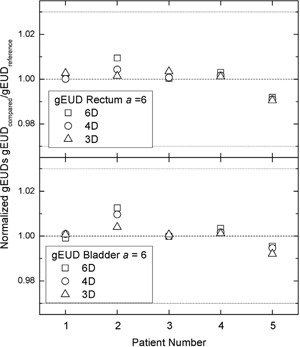
The same comparisons as in Fig. 1 but for rectum and bladder normalized gEUDs.

Figure 2 outlines the comparison among gEUDs for both rectum and bladder. gEUDs were normalized in the same fashion as the DIs. Clearly, the gEUD differences are even smaller than the differences in the DIs, and are well within ±3%.^(^ ^29^ ^)^

### B. Lung plans

**Figure 3:**
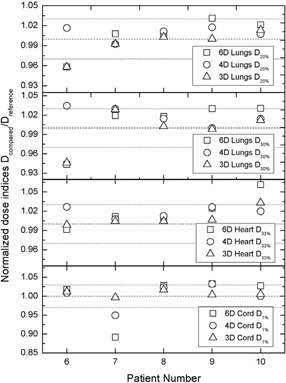
Comparison of normalized DIs for benign lung, spinal cord, and heart in the lung cases.

The same trends for benign lung, heart and spinal cord, presented in Fig. 3 are observed as was observed for rectum DIs (Fig. 1). In case 6, for which the normalized lung DIs $D_{20\%}$ and $D_{30\%}$ are outside ±3%, the actual DIs are less than 13 Gy and 7 Gy, respectively. For patient 10, the actual heart DIs $D_{33\%}$ are less than 20 Gy, while for patient 7, cord $D_{1\%}$ is less than 15 Gy. Therefore, for all cases where the DIs exhibit difference in excess of 3%, the actual doses corresponding to those DIs are well below the OAR tolerances

**Figure 4:**
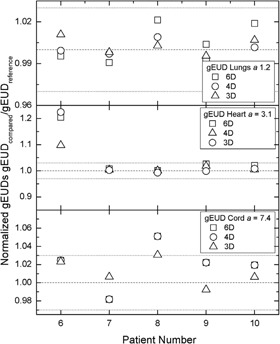
Normalized gEUDs for the lung cases, where lung, spinal cord, and heart cross‐comparisons are shown.

Figure 4 presents the normalized gEUDs. All normalized gEUDs for the lung cases are within ±3%. The heart gEUDs for patient 6 are less than 3 Gy, while the heart gEUDs for the other four patients are of the order of 35 Gy to 45 Gy. The normalized cord gEUDs for patient 8 differ by more than 5%. However, the actual gEUDs are less than 10 Gy compared to actual cord gEUDs for the rest of the lung cases of the order of 25 Gy to 38 Gy.

### C. HN plans

**Figure 5:**
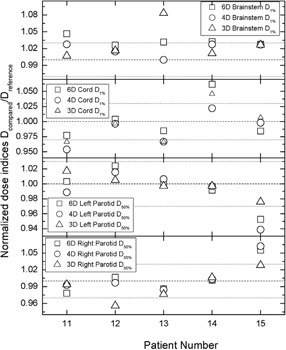
The same as Fig. 1 but for HN DIs for brainstem, spinal cord, and both parotid glands.

Figure 5 outlines the comparison among the different optimization schemes for normalized DIs. For the majority of the DIs the differences are within ±3%. It is evident that for HN plans, more DIs are outside of ±3% and the differences between the references and the standard are larger than in the prostate and the lung cases. For patient 13, the brainstem $D_{1\%}$ for 3° spacing is more than 8% larger than the $D_{1\%}$ for 2° spacing. However, the actual $D_{1\%}$ for 3° spacing is less than 36 Gy, which is well below the optimization objective of 50 Gy. Patient 14's cord $D_{1\%}$ for 6° spacing differs from $D_{1\%}$ for 2□ spacing by ~5% with actual doses of less than 46 Gy, which is again below the 50 Gy optimization objective. Likewise, the right parotid $D_{50\%}$, for 4° spacing in case 15 is less than 12 Gy, which is almost three‐fold lower than the planning objective of 30 Gy to 50% of the parotid volume.

**Figure 6:**
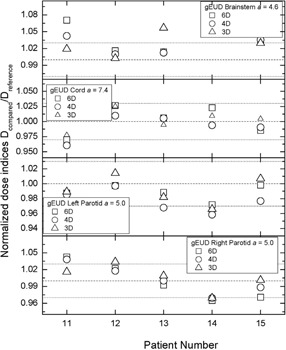
The same as Fig. 2 but for HN gEUDs for brainstem, spinal cord, and both parotid glands.

The normalized gEUD results in Fig. 6 are qualitatively similar to the results for the normalized HN DIs from Fig. 5. The only problematic normalized gEUD is for the right parotid in patient 11 where the actual value for the 6° spacing is almost 40 Gy, while for 2° spacing it is more than 38 Gy, and both gEUDs are among the largest for all five HN cases.

## IV. DISCUSSION

The study compares four different VMAT optimization schemes with as much as three‐fold difference in the number of segments. The presented results indicate that with increasing plan complexity from prostate to HN cases where more targets and more OARs “pull” the optimization objectives in different directions, the dosimetric differences increase with increasing difference in the number of utilized optimization segments. Overall, the differences in majority of the DIs and gEUDs are within ±3%, and therefore would not be clinically significant. In all but one of the cases where the differences are beyond 3%, either the tallied index is of lesser importance (such as gEUDs for spinal cord and brainstem where maximum dose is the important clinical surrogate for complications), or the index is well below the anatomical structure tolerance.

**Figure 7:**
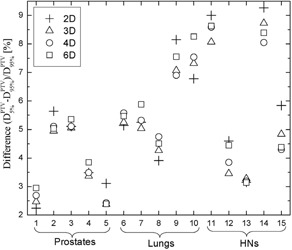
Assessment of dose heterogeneity across the PTV. Presented is PTV $D_{5\%}$ normalized to the prescription dose which is equivalent to PTV $D_{095\%}$.

It has been argued^(^ ^10^ ^)^ that VMAT plans require over ~70 segments for adequate PTV coverage with difference between PTV $D_{95\%}$ and PTV $D_{5\%}$ of less than 6%. The ratio $100\times (D_{5\%}^{PTV}-D_{5\%}^{PTV})/D_{5\%}^{PTV}$ (dose homogeneity index) for all four optimization schemes is presented in Fig. 7. The tallied dose inhomogeneity across the PTV ranges from ~2.5% to ~9.5% which is similar to what other investigators have found.^(^ ^10^ ^)^ Interestingly, Pinnacle TPS generated treatment plans with very similar PTV dose heterogeneity for plans with 61 segments (6° spacing) and 181 segments (2° spacing). This fact is in a marked contrast with the results reported for Varian Eclipse TPS.^(^ ^10^ ^)^ This result is to some extent dependent on issues such as the specifics behind the optimization methodologies. The optimization in this work was performed within a single arc where different spacing between successive control points was specified while, in Eclipse, the plans with different number of segments were created by superimposing several arcs optimized simultaneously. The presented dose inhomogeneity results herein also indicate that with an increasing plan complexity from prostates to HNs, the dose homogeneity across the PTV deteriorates at the expense of target coverage and OAR sparing.

In addition to all DIs, gEUDs and dose homogeneity indices monitor units (MUs) for the different optimization schemes were tallied. The MU differences are presented in Table 2. The normalization was performed with respect to the MUs for the reference (2° spacing) plans. The negative sign indicates that with decreasing number of segments, the VMAT plans require fewer MUs. The differences, however, are not very large and they do not exceed 5% even for the HN cases, and therefore are not likely to influence significantly treatment times and MLC leakage doses.

**Table 2:** Average differences and standard deviations in the fractional MUs among the four different optimization schemes by anatomical site. Quoted are MU differences with respect to the MUs resulting from the reference (2°) optimization. The negative sign denotes that the compared MUs are less than the reference MUs.

	*Prostate (St Dev)*	*Lung (St Dev)*	*HN (St Dev)*
MU difference [3°]	$\begin{array}{c} -1.1\%\;\\ \left(2.5\%\right) \end{array}$	$\begin{array}{c} -1.6\%\;\\ \left(1.5\%\right) \end{array}$	$\begin{array}{c} -3.4\%\;\\ \left(1.5\%\right) \end{array}$
MU difference [4°]	$\begin{array}{c} -1.9\%\;\\ \left(3.1\%\right) \end{array}$	$\begin{array}{c} -3.1\%\;\\ \left(3.3\%\right) \end{array}$	$\begin{array}{c} -4.0\%\;\\ \left(1.2\%\right) \end{array}$
MU difference [6°]	$\begin{array}{c} -3.3\%\;\\ \left(3.7\%\right) \end{array}$	$\begin{array}{c} -4.4\%\;\\ \left(3.8\%\right) \end{array}$	$\begin{array}{c} -4.9\%\;\\ \left(3.0\%\right) \end{array}$

As pointed out in the introduction, VMAT technique is more time efficient than fixed gantry angle IMRT during treatment delivery. In the investigation presented herein the “requested” (from the optimizer) delivery time was always set to be of the order of 10–15 minutes, since the objective of the work was to tally the plan quality dependence on the number of segments rather than the delivery time efficiency. The average delivery times estimated by the optimizer are presented in Table 3. As can be deduced from the data, the delivery times are quite comparable and thereby almost independent of the number of segments. We hypothesize that this result stems in part from the degeneracy of the DVHs,^(^ ^25^ ^,^ ^30^ ^)^ namely that different volumetric dose distributions will correspond to indistinguishable DVHs. Furthermore, the similarity of the estimated delivery times implies that the delivery times are based predominantly on the target shape, plan complexity, and arc length, rather than on the number of delivery segments. Presumably, the estimated delivery times are based on the gantry and MLC leaves constraints and are always minimized with the help of variation in the dose rate.

**Table 3:** Estimated delivery times by anatomical site and angular spacing. The quoted times are in seconds.

	*Prostate*	*Lung*	*HN*
Average delivery time for 2° spacing	76.0	49.8	75.4
Average delivery time for 3° spacing	75.8	49.4	75.2
Average delivery time for 4° spacing	75.6	48.8	75.0
Average delivery time for 6° spacing	75.4	48.6	75.4

VMAT optimization times are directly proportional to the number of segments utilized in the planning process, since the majority of the CPU time is used for dose calculations. The optimization times for a single dynamic arc are of the order of an hour for 2° spacing. Therefore, using coarser segment spacing will result in proportionally shorter treatment planning times.

## V. CONCLUSIONS

The plan quality for all four optimization schemes in this single VMAT optimization study was comparable in the case of Pinnacle's SmartArc implementation. With a three‐fold decrease in the number of optimization control points, the differences in the tallied indices increases. The more complex cases resulted in a more noticeable increase in the differences. The results presented herein indicate that 4° spacing between successive segments in a single arc VMAT plans, optimized with Pinnacle SmartArc, is a very well‐balanced alternative between plan quality and plan complexity.
